# Geriatric Health in India: Concerns and Solutions

**DOI:** 10.4103/0970-0218.43225

**Published:** 2008-10

**Authors:** Gopal K Ingle, Anita Nath

**Affiliations:** Department of Community Medicine, Maulana Azad Medical College, New Delhi - 110002, India

India is in a phase of demographic transition. As per the 1991 census, the population of the elderly in India was 57 million as compared with 20 million in 1951. There has been a sharp increase in the number of elderly persons between 1991 and 2001 and it has been projected that by the year 2050, the number of elderly people would rise to about 324 million.(1) India has thus acquired the label of “an ageing nation” with 7.7% of its population being more than 60 years old. The demographic transition is attributed to the decreasing fertility and mortality rates due to the availability of better health care services. It has been observed that the reduction in mortality is higher as compared with fertility. There has been a sharp decline in the crude death rate from 28.5 during 1951–1961 to 8.4 in 1996; while the crude birth rate for the same time period fell from 47.3 to 22.8 in 1996.(2) Over the past decades, India's health program and policies have been focusing on issues like population stabilization, maternal and child health, and disease control. However, current statistics for the elderly in India gives a prelude to a new set of medical, social, and economic problems that could arise if a timely initiative in this direction is not taken by the program managers and policy makers. There is a need to highlight the medical and socio-economic problems that are being faced by the elderly people in India, and strategies for bringing about an improvement in their quality of life also need to be explored.

## Socio-demographic Profile of the Elderly

According to recent statistics related to elderly people in India, in the year 2001, it was observed that as many as 75% of elderly persons were living in rural areas. About 48.2% of elderly persons were women, out of whom 55% were widows. A total of 73% of elderly persons were illiterate and dependent on physical labor. One-third was reported to be living below the poverty line, i.e., 66% of older persons were in a vulnerable situation without adequate food, clothing, or shelter. About 90% of the elderly were from the unorganized sector, i.e., they have no regular source of income. The number of centenarians in India is about 2,00,000.(1) and India is one of the few countries in the world in which the sex ratio of the aged favors males. This could be attributed to various reasons such as under-reporting of females, especially widows and higher female mortality in different age groups.(3,4)

## Medical and Socio-economic Problems Faced by the Elderly

In India, the elderly people suffer from dual medical problems, i.e., both communicable as well as non–communicable diseases. This is further compounded by impairment of special sensory functions like vision and hearing. A decline in immunity as well as age-related physiologic changes leads to an increased burden of communicable diseases in the elderly. The prevalence of tuberculosis is higher among the elderly than younger individuals. A study of 100 elderly people in Himachal Pradesh found that most of the patients came from a rural background. They were also smokers and alcoholics.(5) It is shown that among the population over 60 years of age, 10% suffer from impaired physical mobility and 10% are hospitalized at any given time, both proportions rising with increasing age. In the population over 70 years of age, more than 50% suffer from one or more chronic conditions.(6) The chronic illnesses usually include hypertension, coronary heart disease, and cancer. According to Government of India statistics, cardiovascular disorders account for one-third of elderly mortality. Respiratory disorders account for 10% mortality while infections including tuberculosis account for another 10%. Neoplasm accounts for 6% and accidents, poisoning, and violence constitute less than 4% of elderly mortality with more or less similar rates for nutritional, metabolic, gastrointestinal, and genito-urinary infections.(7)

An Indian Council of Medical Research (ICMR) report on the chronic morbidity profile in the elderly states that hearing impairment is the most common morbidity followed by visual impairment.(8) However, different studies show varied results in the morbidity pattern. A study conducted in the rural area of Pondicherry reported decreased visual acuity due to cataract and refractive errors in 57% of the elderly followed by pain in the joints and joint stiffness in 43.4%, dental and chewing complaints in 42%, and hearing impairment in 15.4%. Other morbidities were hypertension (14%), diarrhea (12%), chronic cough (12%), skin diseases (12%), heart disease (9%), diabetes (8.1%), asthma (6%), and urinary complaints (5.6%).(9) A similar study that had been conducted among 200 elderly people in rural and urban areas of Chandigarh in Haryana observed that as many as 87.5% had minimal to severe disabilities. The most prevalent morbidity was anemia, followed by dental problems, hypertension, chronic obstructive airway disease (COAD), cataract, and osteoarthritis.(10) A study on ocular morbidities among the elderly population in the district of Wardha found that refractive errors accounted for the highest number (40.8%) of ocular morbidities, closely followed by cataract (40.4%) while other morbidities included aphakia (11.1%), pterygium (5.2%), and glaucoma (3.1%).(11) In a community based study conducted in Delhi among 10,000 elderly people, it was found that problems related to vision and hearing topped the list, closely followed by backache and arthritis.(12)

Elderly people who belong to middle and higher income groups are prone to develop obesity and its related complications due to a sedentary lifestyle and decreased physical activity.(13) In a study conducted among 206 elderly persons attending the Geriatric Clinic at a tertiary care hospital in Delhi, about 34% of the men and 40.3% of the women were obese respectively.(14)

Elderly people are highly prone to mental morbidities due to ageing of the brain, problems associated with physical health, cerebral pathology, socio-economic factors such as breakdown of the family support systems, and decrease in economic independence. The mental disorders that are frequently encountered include dementia and mood disorders. Other disorders include neurotic and personality disorders, drug and alcohol abuse, delirium, and mental psychosis.(15)

The rapid urbanization and societal modernization has brought in its wake a breakdown in family values and the framework of family support, economic insecurity, social isolation, and elderly abuse leading to a host of psychological illnesses. In addition, widows are prone to face social stigma and ostracism.(16) The socio-economic problems of the elderly are aggravated by factors such as the lack of social security and inadequate facilities for health care, rehabilitation, and recreation. Also, in most of the developing countries, pension and social security is restricted to those who have worked in the public sector or the organized sector of industry.(17) Many surveys have shown that retired elderly people are confronted with the problems of financial insecurity and loneliness.(18,19)

The 60^th^ National Sample Survey (January–June 2004) collected data on the old age dependency ratio. It was found to be higher in rural areas (125) than in urban areas (103). With regard to the state of economic development, a higher number of males in rural areas, 313 per 1000, were fully dependent as compared with 297 per 1000 males in urban areas. For the aged female, an opposite trend was observed (706 per 1000 for females in rural areas compared with 757 for females in urban areas).(20) Overall 75% of the economically dependent elderly are supported by their children and grandchildren. Despite this, the elderly still tend to suffer from psychological stress as was found in a survey conducted for a middle class locality in New Delhi.(21) Over 81% of the elderly confessed to having increasing stress and psychological problems in modern society, while 77.6% complained about mother-in-law/daughter-in-law conflicts being on the increase.

The elderly are also prone to abuse in their families or in institutional settings. This includes physical abuse (infliction of pain or injury), psychological or emotional abuse (infliction of mental anguish and illegal exploitation), and sexual abuse. A study that examined the extent and correlation of elder mistreatment among 400 community-dwelling older adults aged 65 years and above in Chennai found the prevalence rate of mistreatment to be 14%. Chronic verbal abuse was the most common followed by financial abuse, physical abuse, and neglect. A significantly higher number of women faced abuse as compared with men; adult children, daughters-in-law, spouses, and sons-in-law were the prominent perpetrators.(22)

The Central and State governments have already made efforts to tackle the problem of economic insecurity by launching policies such as the National Policy on Older Persons, National Old Age Pension Program, Annapurna Program, etc. However, the benefits of these programs have been questioned several times in terms of the meager budget, improper identification of beneficiaries, lengthy procedures, and irregular payment.(23)

## Strategies to Improve the Quality-of-Life of the Elderly: The Role of the Health Care System

With a brief overview of the health and socio-economic challenges that are being faced by the elderly population in India, the following strategies may be explored by the program managers of the public health care system to bring about improvement in the quality-of-life of the geriatric population.

At present, most of the geriatric out patient department (OPD) services are available at tertiary care hospitals. Also, most of the government facilities such as day care centers, old age residential homes, and counseling and recreational facilities are urban based. A study conducted to assess the unmet needs of the geriatric population in rural Meerut observed that as many as 46.3% of the study participants were unaware of the availability of any geriatric services near their residence and 96% had never used any geriatric welfare service. About 59% of them stated that the nearest government facility was 3 kilometers from their homes.(19)

Since 75% of the elderly reside in rural areas, it is mandatory that geriatric health care services be made a part of the primary health care services. This calls for specialized training of Medical Officers in geriatric medicine. Also, factors such as a lack of transport facilities and dependency on somebody to accompany an elderly person to the health care facility impede them from accessing the available health services. Thus, peripheral health workers and community health volunteers should also be trained to identify and refer elderly patients for timely and proper treatment. An ICMR task force project, which was known as “Health Care of the Rural Aged”, conducted in the Primary Health Center area near Madurai found this strategy to be beneficial.(24)

In difficult to access areas, screening camps for cataract and non-communicable diseases and mobile clinics could play a significant role in reaching out to the elderly population. Advocacy with non-governmental organizations (NGOs), charitable organizations, and faith-based organizations could play an important role in this aspect. Premier NGOs like Help Age India have already been organizing screening camps and providing Mobile Medical Units in rural and difficult to access areas.

Ensuring good quality geriatric health care services at the primary level would greatly help in improving the utilization rates of the available health services. Health care services should be based on the “felt needs” of the elderly population. This would involve a comprehensive baseline morbidity survey and functional assessment in health areas that are perceived to be important to them. This should be transformed into a community database that would help to prioritize interventions and allocate finances accordingly. The felt needs may vary depending upon gender; socio-economic status as well as differences would exist in the rural and urban areas. Until now, secondary prevention strategies in the form of screening and early management and tertiary care in the form of rehabilitation have been given more importance as compared with primary prevention by the geriatric health care services. Projections made by the World Health Organization (WHO) suggest that by 2015 deaths from chronic diseases such as cancer, hypertension, cardiovascular diseases, and diabetes will increase by 17 percent, from 35 million to 41 million.(25) This calls for a multi-pronged intervention program that should be viable and easily monitored.(26)

An ideal preventive health package should include various components such as knowledge and awareness about disease conditions and steps for their prevention and management, good nutrition and balanced diet, and physical exercise. For the promotion of a positive mindset and to create a feeling of well being, meditation, prayer, and strategies for motivation should also be included.(27)

Capacity building may be done for different groups of health personnel. Training of Medical Officers and peripheral health workers has been discussed above. Besides this, an entirely distinct team of health providers known as “Community Geriatric Health Workers” may be trained to provide home care to the disabled elderly population. This strategy has been demonstrated to be successful in a community based project in Cochin, known as “Urban Community Dementia Services” wherein these health workers provide home-based care as well as care in day care centers.(28)

According to the findings of the 60^th^ NSSO Round, the proportion of aged persons who cannot move and are confined to their bed or home ranges from 77 per 1000 in urban areas to 84 per 1000 in rural areas.(20) Strengthening the elderly in the process of self-help can be done by means of physical, psychosocial, and vocational rehabilitation. Rehabilitation includes (i) provision of visual aids/mobility aids at geriatric health facilities, (ii) the availability of physiotherapy services, and (iii) imparting health education about staying mobile and providing practical tips. Rehabilitation comprises of provisions for counseling services wherein older persons can benefit from psychological assistance in the face of stressful life events, interpersonal conflicts, and changes imposed by ageing.(29) Under rehabilitation, health care facilities should aim for holistic development by organizing training workshops in accordance with the skills of the elderly. This calls for advocacy with NGOs and charitable organizations. Opportunities for employment should be provided simultaneously.

Also, capacity building of the community leaders is essential for the success of community-based geriatric and rehabilitative health services. Community leaders can play an important role in identifying the felt needs of the elderly and in resource generation.

Among the secondary level health facilities, which mainly include the district hospitals, sub-district, and medium-size private hospitals, it is seen that India has about 12,000 hospitals with 7 lakh beds. Most of these beds are under the public sector.(30) The need of the hour is to set up geriatric wards that would fulfill the specific needs of the geriatric population by provision of distinct OPD services. Providing screening services as well as curative and rehabilitative services and convalescent homes to provide long-term care, which may be a part of designated hospitals, is also a priority.

At the tertiary care level, which comprises of super specialty and medical college hospitals, there needs to be provision of geriatric wards and separate OPDs. A"multi-disciplinary team" specifically trained to meet the needs of the geriatric population need to be created. This team would be comprised of a physician, psychiatrist, orthopaedician, diabetologist, gynecologist, cardiologist, urologist, eye surgeon, psychologist, physiotherapist, dietician, dentist, and nurses trained in geriatric medicine. Elderly patients from poor and low income facilities should be supplied with free or reasonably priced treatment through public-private partnership.

Day care hospitals could play an important role in providing close supervision and follow-up of patients with chronic diseases. Moreover, the cost of a day care centre is comparatively less than that of a nursing home. India has very few hospices that can provide terminal patient care. Hospices should be set up at the district level. NGOs, charitable organizations, and faith-based organizations could play an important role in this area.(30)

Professional training in Geriatrics and Gerontology needs to be promoted. Few universities, for example, the Indira Gandhi National Open University, offer a Post-graduate diploma in Geriatric Medicine. There is a need to give emphasis to geriatric medicine in undergraduate medical as well as paramedical courses. Geriatric dentistry should also be developed as a separate, independent specialty at the post-graduate level.(31)

Research in Geriatrics and Gerontology needs to be further encouraged. An ICMR Workshop on “Research and Health Care Priorities in Geriatric Medicine and Ageing” recommended that research be conducted in areas such as the evaluation of the nutritional and functional status of the elderly, common chronic and neuro-degenerative disorders like Alzheimer's disease, cardiovascular disorders, depression, etc., basic sciences, dealing with the process of ageing, pharmacokinetics and pharmacodynamics of drugs, health system research and research in alternative medicine.(32) Certain lacunae in the field of research on gerontology have been identified, such as the lack of attention given towards the aged in rural India, failure to view elderly people as active participants in the economy, the perception of older persons as being mere recipients of social welfare services, and a lack of focus on policy recommendations.(33)

In conclusion, current trends in demographics coupled with rapid urbanization and lifestyle changes have led to an emergence of a host of problems faced by the elderly in India. Although this paper has mainly focused on the medical problems of the elderly and strategies for improving health care services, it must be remembered that improving the quality-of-life of the elderly calls for a holistic approach and concerted efforts by the health and health-related sectors.
